# Human Beta Defensin 2 Ameliorated Alcohol-Associated Liver Disease in Mice

**DOI:** 10.3389/fphys.2021.812882

**Published:** 2022-01-27

**Authors:** Jeffrey B. Warner, Ida S. Larsen, Josiah E. Hardesty, Ying L. Song, Dennis R. Warner, Craig J. McClain, Rui Sun, Zhongbin Deng, Benjamin A. H. Jensen, Irina A. Kirpich

**Affiliations:** ^1^Division of Gastroenterology, Hepatology, and Nutrition, Department of Medicine, University of Louisville, Louisville, KY, United States; ^2^Department of Pharmacology and Toxicology, University of Louisville School of Medicine, Louisville, KY, United States; ^3^Québec Heart and Lung Institute (IUCPQ), Faculty of Medicine, Laval University, Québec city, QC, Canada; ^4^University of Louisville Alcohol Center, University of Louisville School of Medicine, Louisville, KY, United States; ^5^University of Louisville Hepatobiology and Toxicology Center, University of Louisville School of Medicine, Louisville, KY, United States; ^6^Robley Rex Veterans Medical Center, Louisville, KY, United States; ^7^James Graham Brown Cancer Center, University of Louisville, Louisville, KY, United States; ^8^Department of Surgery, University of Louisville, Louisville, KY, United States; ^9^Department of Biomedical Sciences, Faculty of Health and Medical Sciences, University of Copenhagen, Copenhagen, Denmark

**Keywords:** alcohol, defensin, gut-liver axis, intestine, ALD, immunomodulation, microbiota

## Abstract

Alcohol-associated liver disease (ALD) is a prevalent liver disorder and significant global healthcare burden with limited effective therapeutic options. The gut-liver axis is a critical factor contributing to susceptibility to liver injury due to alcohol consumption. In the current study, we tested whether human beta defensin-2 (hBD-2), a small anti-microbial peptide, attenuates experimental chronic ALD. Male C57Bl/6J mice were fed an ethanol (EtOH)-containing diet for 6 weeks with daily administration of hBD-2 (1.2 mg/kg) by oral gavage during the final week. Two independent cohorts of mice with distinct baseline gut microbiota were used. Oral hBD-2 administration attenuated liver injury in both cohorts as determined by decreased plasma ALT activity. Notably, the degree of hBD-2-mediated reduction of EtOH-associated liver steatosis, hepatocellular death, and inflammation was different between cohorts, suggesting microbiota-specific mechanisms underlying the beneficial effects of hBD-2. Indeed, we observed differential mechanisms of hBD-2 between cohorts, which included an induction of hepatic and small intestinal IL-17A and IL-22, as well as an increase in T regulatory cell abundance in the gut and mesenteric lymph nodes. Lastly, hBD-2 modulated the gut microbiota composition in EtOH-fed mice in both cohorts, with significant decreases in multiple genera including *Barnesiella*, *Parabacteroides*, *Akkermansia*, and *Alistipes*, as well as altered abundance of several bacteria within the family Ruminococcaceae. Collectively, our results demonstrated a protective effect of hBD-2 in experimental ALD associated with immunomodulation and microbiota alteration. These data suggest that while the beneficial effects of hBD-2 on liver injury are uniform, the specific mechanisms of action are associated with baseline microbiota.

## Introduction

Alcohol consumption is a major global health burden and key etiological factor in the development of alcohol-associated liver disease (ALD), which is responsible for nearly half of liver cirrhosis deaths (Seitz et al., 2018). ALD is a spectrum of liver pathologies ranging from hepatic steatosis to inflammation, fibrosis, and hepatocellular carcinoma (Seitz et al., 2018). In addition to the liver, alcohol also induces alterations in other organs, such as the intestine. In both the liver and the gut, alcohol consumption has marked immunomodulatory effects on innate and adaptive immunity, including alterations in the anti-inflammatory response, increased pro-inflammatory cytokine production, and impaired antigen presentation and T cell responses (Szabo and Saha, 2015). Additionally, alcohol exposure can impair gut barrier function (Kirpich et al., 2013; Bishehsari et al., 2017; Warner et al., 2019), alter intestinal anti-microbial defense (Szabo and Saha, 2015), and induce changes in the gut microbiota composition and function, thus contributing to alcohol-induced liver pathology *via* the gut-liver axis (Engen et al., 2015; Sarin et al., 2019). Importantly, the host gut microbiota is not only a target of alcohol toxicity but also may modulate susceptibility to alcohol-associated liver and intestinal damage (Llopis et al., 2016; Ferrere et al., 2017).

Despite the high prevalence of ALD, effective therapeutic options for this disease are limited. One group of compounds that may be beneficial for alcohol-associated liver and gut injury is anti-microbial peptides (AMPs), molecules which serve as a first line of innate defense against invading pathogens, primarily on mucosal surfaces (Zhao and Lu, 2014; Hendrikx and Schnabl, 2019). Several previous reports support favorable effects of various AMPs, such as regenerating islet-derived lectins (e.g., Reg3β and Reg3γ) (Wang et al., 2016) and cathelicidin-related AMP (CRAMP) (Li et al., 2020), which ameliorate ALD by mechanisms including reduction of bacterial colonization of mucosal surfaces, prevention of bacteria/bacterial product translocation through the gut, and abrogation of hepatic inflammasome activation, among others. Similarly, a recent study demonstrated that a functional knockout of human alpha defensins exacerbated bacterial product translocation and subsequent liver injury (Zhong et al., 2020). Therapeutically, alpha defensin 5 administration ameliorated the above-mentioned effects, suggesting a beneficial role for this molecule in treating ALD (Zhong et al., 2020). Of particular interest in the current study are the structurally similar human beta defensins (hBDs), a subclass of AMPs consisting of six identified isoforms (numbered hBD-1–6) (Yamaguchi et al., 2002) that are secreted by leukocytes and epithelial cells in the intestine, skin, lungs, liver, and other organs (Harada et al., 2004; Pazgier et al., 2006). While some human beta defensins are constitutively expressed, others are induced following exposure to microbial pathogens (e.g., hBD-2–4). Of the induced hBDs, evidence suggests hBD-2 is a highly up-regulated isoform in diseases associated with inflammation, such as inflammatory bowel disease (Wehkamp et al., 2002) and colitis (Rahman et al., 2011), where inflammatory signals and bacterial products induce its expression. hBD-2 exerted favorable effects in various disease models through its dual anti-microbial and immunomodulatory functions which are mediated by either direct binding to bacteria through lipopolysaccharide and pore complex formation, or *via* host cell surface receptors such as CCR2, CCR6, and TLR4 (Semple and Dorin, 2012). For example, hBD-2 has been shown to attenuate experimental colitis by a CCR2-mediated immunomodulatory mechanism, highlighting its potential therapeutic benefits (Koeninger et al., 2020). Still, the effects of hBD-2 in ALD remain largely unknown.

In the current study, we investigated whether hBD-2 is beneficial in experimental ALD due to chronic EtOH consumption. The study was performed using two independent cohorts of mice with distinct gut microbiota to determine the impact of the microbiome on the effects of hBD-2. Our data demonstrated that hBD-2 ameliorated liver injury in both mouse cohorts. Mechanistically, hBD-2 exerted favorable, but differential effects in these cohorts including immunomodulatory changes in the liver and the gut, suggesting that the mechanisms contributing to the beneficial effects of hBD-2 are broad and gut microbiota-dependent.

## Materials and Methods

### Animal Model and Human Beta Defensin 2 Administration

**FIGURE 1:**
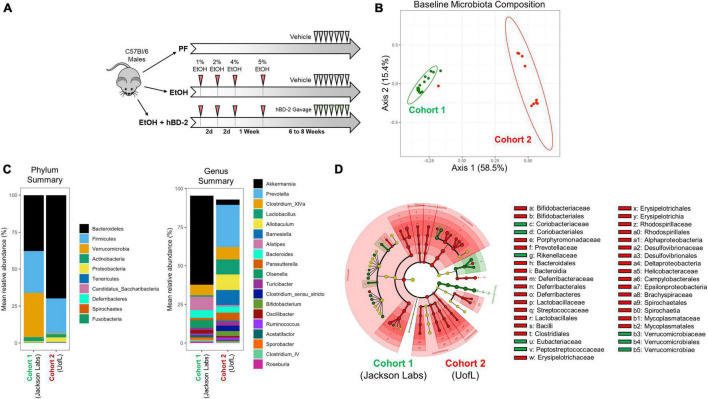
Experimental design and gut microbiota differences between cohorts at baseline. **(A)** Schematic representation of study design. Two cohorts of male C57BL/6J mice were subjected to chronic EtOH feeding for 6–8 weeks in three experimental groups: pair-fed (PF), EtOH-fed + vehicle, EtOH-fed + hBD-2. hBD-2 was administered daily by oral gavage on the final 7 days of the EtOH feeding. **(B)** Principal coordinate analysis using Bray-Curtis distance from fecal 16S sequencing data showing independent microbiota between the two cohorts at baseline, before EtOH feeding began. Individual data points are shown as points with indicated cohort 50% confidence interval. PERMANOVA *p* = 0.001 between the cohorts. **(C)** Phylum- and genus-level summary of mean relative bacterial abundance between the cohorts at baseline. **(D)** LEfSe cladogram generated from fecal 16S sequencing data showing significant differences in taxonomical groups of bacteria at baseline associated with Cohort 1 (green) or Cohort 2 (red).

All animal studies were approved by and executed within the guidelines of the University of Louisville (UofL) Institutional Animal Care and Use Committee and the NIH Office of Laboratory Animal Welfare Guidelines. The study was performed with two independent cohorts of mice, Cohort 1 (referred to as “Jackson Labs Cohort,” C57BL/6J mice directly purchased from Jackson Laboratories, Bar Harbor, ME) and Cohort 2 (referred to as “UofL Cohort,” second to third generation C57BL/6J offspring generated at the UofL Animal facility from breeder pairs purchased from Jackson Laboratories). Mice were maintained in micro-isolator cages on a 12-h light/dark cycle. 8- to 10-week-old male mice were subjected to a chronic ethanol (EtOH) feeding protocol. In this feeding paradigm, mice were first adjusted to a control liquid diet during a one-week acclimation phase, then ramped up to a 5% (w/v) EtOH diet as follows: 2 days on 1%, 2 days on 2%, one week on 4%, then 5% EtOH for the remainder of the study, for a total of 6–8 weeks of EtOH feeding. The diets, F1258 [EtOH] and F1259 [control] were purchased from Bio-Serv (Flemington, NJ). hBD-2 (a generous gift from Defensin Therapeutics, Copenhagen, Denmark) was administered by oral gavage once daily for the 7 days prior to sacrifice at a dose of 1.2 mg/kg. This dose was chosen based on its therapeutic efficacy and low toxicity in mice with different pathologies, such as experimental colitis (Koeninger et al., 2020) and asthma (Borchers et al., 2021; Pinkerton et al., 2021). As per the manufacturer, the hBD-2 solution had a purity of 98.6% determined by Ultra Performance Liquid Chromatography. The crystal structure of hBD-2 has been previously reported (Hoover et al., 2000; Sawai et al., 2001). Mice that did not receive hBD-2 were administered an equivalent volume of phosphate-buffered saline by oral gavage as vehicle control. Cohort 1 included PF, EtOH + vehicle, and EtOH + hBD-2 experimental groups. To confirm the effects of hBD-2 on EtOH-mediated changes, Cohort 2 included only EtOH + vehicle and EtOH + hBD-2 experimental groups. At the conclusion of the experiment, animals were euthanized by deep anesthesia with ketamine/xylazine prior to blood draw and tissue/organ harvest. A scheme of the experimental design is presented in the Figure 1A.

### Plasma Alanine Aminotransferase Measurement

Plasma alanine aminotransferase (ALT) levels were measured as a biomarker of liver injury using the ALT/GPT reagent as per manufacturer’s instructions (Thermo Fisher, Waltham, MA).

### Hepatic Triglyceride Analysis

Liver triglyceride levels were measured as previously described (Kirpich et al., 2011) using Thermo Fisher reagents.

### Blood Alcohol Concentration Measurement

Blood alcohol concentration was measured in undiluted plasma samples with the EnzyChrom Ethanol Assay Kit as per the manufacturer’s instructions (BioAssay Systems, Hayward, CA).

### Histopathological and Immunohistochemical Analysis of Liver Tissue

Formalin-fixed, paraffin-embedded samples of liver tissue were cut to 5 μm thickness, then stained with either hematoxylin & eosin (H&E staining) to evaluate gross hepatic pathology, Oil Red O to evaluate hepatic steatosis (ORO, Electron Microscopy Sciences, Hatfield, PA), chloroacetate esterase (CAE, Sigma Aldrich, St. Louis, MO) or myeloperoxidase (MPO, R&D Systems, Minneapolis, MN) for analysis of neutrophil infiltration. Terminal deoxynucleotidyl transferase dUTP nick-end labeling (TUNEL) was used for analysis of hepatocellular death (ApopTag Peroxidase *in situ* Apoptosis Detection Kit, Millipore, Burlington, MA). Quantification of CAE, MPO, and TUNEL staining was performed *via* light microscopy by blindly quantifying positive cells in 10–20 random digital images (200 × magnification) per liver section (*n* = 5–10 sections per group) by two independent investigators. Positive cells were then averaged between images to obtain an average per mouse. Quantification of ORO staining was performed in a similar manner, except using the color threshold feature of ImageJ software to determine percentage of the image positive for ORO staining.

### RNA Isolation and Real-Time Quantitative PCR

Total RNA was isolated from the liver and ileum tissue using Trizol (Thermo Fisher). Genomic DNA contamination was removed with the TURBO DNA-free kit (Thermo Fisher). cDNA was synthesized from 1 μg of total RNA with qScript cDNA Supermix (Quanta Biosciences, Beverly, MA). 10 ng cDNA was then used for each reverse transcription qPCR reaction using PerfeCTa SYBR Green Fast Mix (Quanta Biosciences) in a BioRad CFX384 qPCR instrument (Hercules, CA). qPCR data was analyzed by the ΔΔCt method (Livak and Schmittgen, 2001). Primer sequences are presented in Table 1.

**TABLE 1:** qPCR primer sequences (5′–3′).

Target	Forward	Reverse
*18S*	CTCAACACGGGAAACCTCAC	CGCTCCACCAACTAAGAACG
*Il-17a*	GGAGAGCTTCATCTGTGTCTCTGA	GAAGTCCTTGGCCTCAGTGTT
*Il-22*	ATCAGCTCAGCTCCTGTCACAT	TCCAGTTCCCCAATCGCCTT
*Cxcl1*	GGAAGTGTGATGACTCAGGTTTGC	GATGGTTCCTTCCGGTGGTTTCTTC
*Mcp1*	GGCTCAGCCAGATGCAGT	TGAGCTTGGTGACAAAAACTACAG
*Mip2α*	GCGCCCAGACAGAAGTCATA	TCCAGGTCAGTTAGCCTTGC
*Zo-1*	TGGGAACAGCACACAGTGAC	GCTGGCCCTCCTTTTAACAC
*Zo-2*	CGCTGATGGCTTGCTTCA	AACCTTCCGGGGTCTCTTG
*Occludin*	ACCCGAAGAAAGATGGATCG	CATAGTCAGATGGGGGTGGA

### Immunoassay of Cytokines in Liver and Ileum Tissues

Liver and ileum tissue samples were homogenized in 250 μL PBS plus 0.05% Tween-20, protease and phosphatase inhibitors (Halt, Thermo Fisher), 0.5 M EDTA, 0.5 M EGTA, and 0.25 M sucrose using 2.8 mm ceramic beads (Biotage, Charlotte, NC) by shaking at 50 cycles/second for 1 min with a Qiagen TissueLyser LT instrument (Qiagen, Germantown, MD). Insoluble material was removed by centrifugation at 19,000 × *g* for 10 min. Protein concentrations were determined by the bicinchoninic acid assay (BCA) (Pierce BCA protein assay kit, Thermo Fisher), and then 300 μg protein for ileum samples or 600 μg protein for liver samples was analyzed using the MesoScale Discovery (MesoScale Discovery, Rockville, MD) V-PLEX TH17 Panel 1 kit (catalog number K15085D). Data were collected using the MESO Sector S 600 instrument and reduced with Discovery Workbench (v 4.0) software (MesoScale Discovery).

### Intestine and Mesenteric Lymph Node Immune Cell Isolation and Flow Cytometry Analysis

Immune cell isolation from small intestines (intraepithelial lymphocytes [IELs] and lamina propria leukocytes [LPLs]), large intestines (IELs and LPLs), liver, and mesenteric lymph nodes (MLNs) was performed by a previously described procedure (Chu et al., 2020). Briefly, to isolate small intestine and large intestine IELs and LPLs, the intestine was cut into 0.5 cm pieces and incubated in HBSS with 5 mM EDTA for 30 min at 37°C with 180 RPM shaking. IELs were recovered in the supernatant. Large intestine pieces were then incubated in an additional HBSS solution with 0.5 mg/mL DNase I (Roche, Indianapolis, IN) and 1 mg/mL collagenase type IV (Worthington, Lakewood, NJ), then passed through a 100 μm strainer. Lamina propria lymphocytes were recovered at the interface between 40 and 72% Percoll layer (GE Healthcare, Chicago, IL). For liver and MLNs, tissue was mechanically homogenized with a rubber-tipped syringe and passed through a 70 μm strainer. Immune cells were labeled using the FOXP3/Transcription Factor Staining Buffer Set as per manufacturer’s instructions (Thermo Fisher). 1/100 dilutions of APC-labeled anti-CD4 (RM4-5) and either FITC-labeled FOXP3 (FJK-16s), PE-labeled anti-IFN gamma (XMG1.2) or PerCP-Cyanine5.5-labeled anti-IL17 (eBio17B7) were used to label T regulatory cells, Th1 cells, and Th17 cells, respectively. The same dilution of APC-labeled anti-B220 (RA3-6B2) and either FITC-labeled anti-IgM (II/41) or PE-labeled anti-IgA (mA-6E1) were used to label B cells. All antibodies were obtained from Thermo Fisher. Data were collected using a BD FACSCanto II flow cytometer and analyzed with FlowJo software v10.7 (Becton Dickinson, Franklin Lake, NJ).

### Fecal Microbiota Composition Analysis and Bioinformatics

Changes in fecal microbiota were analyzed by 16S rRNA gene amplicon sequencing. Fresh feces were collected at baseline prior to EtOH feeding as well as at the termination of the study, and DNA was extracted using the NucleoSpin 96 soil kit (Macherey-Nagel, Düren, Germany) following the manufacturer’s instructions. Baseline samples were processed as previously described (Jensen et al., 2021). Briefly, 30 ng DNA and 16S rRNA fusion primers were added for PCR followed by purification with Agencourt AMPure XP beads (Beckman Coulter, Indianapolis, IN). Library size and concentration was measured by Agilent 2100 Bioanalyzer and sequenced on the HiSeq platform (Illumina, San Diego, CA) according to insert size. Raw sequences were filtered to obtain the high-quality clean data, then overlapping sequences were merged to tags and further clustered to operational taxonomic units. Taxonomic classifications of operational taxonomical units were annotated using the Ribosomal Database Project database. The 16S rRNA genes of endpoint samples were amplified using primers for the V3-V4 region with Illumina adaptors (S-D-Bact-0341-b-S-17: *5′-TCGTCGGCAGCGTCAGATGTGTATAAGAGACAGCCTACGG GNGGCWGCAG-3′ and* S-D-Bact-0785-a-A-21: *5′-GTCTCGT GGGCTCGGAGATGTGTATAAGAGACAGGACTACHVGGGTA TCTAATCC-3′)* (Klindworth et al., 2013). Indices were added in a second PCR using the Nextera XT Index Kit V2 (Illumina) followed by purification with Agencourt AMPure XP beads (Beckman Coulter). The library was sequenced using a MiSeq desktop sequencer (Illumina). Raw sequences were filtered, and amplicon sequencing variants (ASVs) were generated with usearch and taxonomic classification of ASVs were annotated using the Silva database (Quast et al., 2013).

Analysis of microbiota composition was carried out in R and R Studio using the phyloseq package (McMurdie and Holmes, 2013). Permutational analysis of variance (PERMANOVA) on principal coordinate analyses (PCoAs) was carried out using the vegan package (Oksanen et al., 2020). Linear discriminant analysis Effect Size (LEfSe) was carried out using the public Galaxy server (Afgan et al., 2018). Linear mixed-effects modeling was performed using the lme4 package (Bates et al., 2015) at ASV level using a cutoff of ASVs with an overall relative abundance of minimum 0.1%. The ggplot2 package was used for visualization of the data.

### Statistical Analysis

Data are reported as the mean ± standard error of the mean (SEM). For data that fit a normal distribution, differences between two groups were evaluated using the unpaired two-tailed Student’s *t*-test or one-way analysis of variance (ANOVA) followed by Tukey’s multiple comparisons test for three groups. For data that did not fit a normal distribution (as determined by the Shapiro-Wilk normality test), differences between two groups were analyzed by Mann-Whitney *U* test or Kruskal Wallis *H* test followed by Dunn’s multiple comparisons test for three or more groups. Differences were considered statistically significant at a *p* value of less than 0.05. Statistical analyses were carried out using GraphPad Prism 8.1 software (GraphPad, La Jolla, CA).

## Results

### Human Beta Defensin 2 Ameliorated Experimental Alcohol-Associated Liver Disease in Two Independent Cohorts of Mice

To determine the effects of hBD-2 in experimental chronic ALD, we used a chronic EtOH feeding animal model with hBD-2 administered by oral gavage in a treatment paradigm (Figure 1A). This model recapitulates many features of human ALD including mild liver injury and steatosis (Ghosh Dastidar et al., 2018). The study was performed with two independent cohorts of mice: Cohort 1 (the Jackson Laboratories cohort) and Cohort 2 (the UofL cohort) with distinct baseline gut microbiota compositions (Figures 1B–D). Specifically, at the phylum level, Cohort 1 had greater abundance of Verrucomicrobia and lower abundance of Spirochaetia than Cohort 2, and at the genus level, Cohort 1 had greater abundance of *Akkermansia* and *Alistipes*, whereas Cohort 2 had greater abundance of *Prevotella* and *Lactobacillus*, among numerous other differences (Figures 1C,D). Over the course of the feeding protocol, there were no significant differences in food consumption or body weights (Table 2). Similarly, at the conclusion of the experiment, there were no significant changes in liver/body weight or fat/body weight ratios between groups in each cohort or between cohorts (Table 2).

**TABLE 2:** Metabolic characteristics of the experimental mice.

	Cohort 1			Cohort 2	
Characteristic	PF	EtOH	EtOH + hBD-2	EtOH	EtOH + hBD-2
	*n* = 6	*n* = 6	*n* = 6	*n* = 8	*n* = 10
Initial BW (g)	26.15 ± 0.31	26.05 ± 0.33	26.16 ± 0.45	26.69 ± 1.10	25.98 ± 0.41
Final BW (g)	33.79 ± 0.92	32.06 ± 0.33	33.75 ± 1.75	27.51 ± 1.19	28.44 ± 0.85
Liver/BW Ratio (%)	3.894 ± 0.21	4.364 ± 0.14	4.487 ± 0.13	3.755 ± 0.07	3.793 ± 0.07
Fat/BW Ratio (%)	2.861 ± 0.19	2.844 ± 0.20	2.722 ± 0.28	2.358 ± 0.24	2.842 ± 0.28
Food consumption (g per day per mouse)	*	13.21 ± 0.25	12.84 ± 0.28	13.27 ± 0.29	13.50 ± 0.28
**Biochemical measurements**					
Liver triglycerides (mg per g liver)	45.57 ± 6.416	35.91 ± 2.717	41.03 ± 10.470	71.53 ± 8.389	54.49 ± 4.720
Plasma LPS (EU/mL)	0.475 ± 0.048	0.393 ± 0.028	0.478 ± 0.042	0.300 ± 0.025	0.660 ± 0.217
Blood alcohol concentration (mM)	**	1.458 ± 0.172	1.673 ± 0.100	2.026 ± 0.425	1.595 ± 0.157

*Values are expressed as mean ± SEM.*

*PF mice consume the same amount of food as EtOH-fed mice (pair-feeding paradigm).

**Blood alcohol concentration was not measured in PF mice.

**FIGURE 2:**
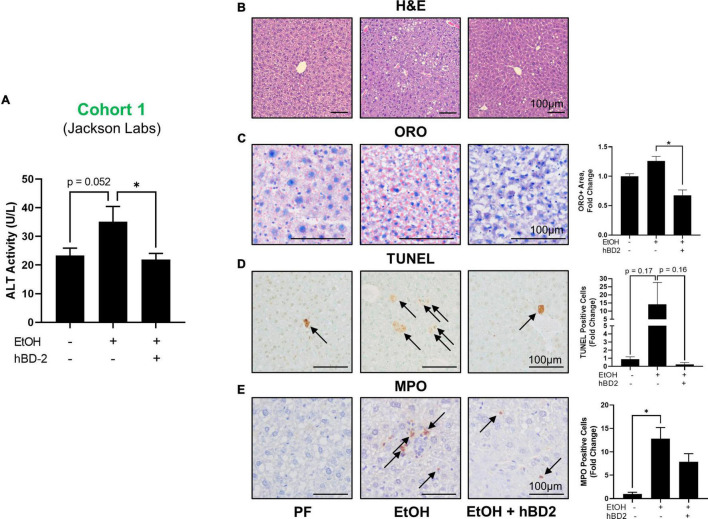
Human beta defensin 2 ameliorated liver injury in Cohort 1. **(A)** ALT activity levels. **(B–E)** Representative digital micrographs of H&E-stained liver sections, ORO-stained liver sections, TUNEL-stained liver sections, and MPO immunohistochemistry with quantitation of ORO+ area, TUNEL+ cells, and MPO+ neutrophils shown to the right. H&E images were captured at 100 × magnification; all other images were captured at 200 × magnification. Scale bar represents 100 μm in each panel. Data are reported as mean ± standard error of the mean. *p < 0.05.

**FIGURE 3:**
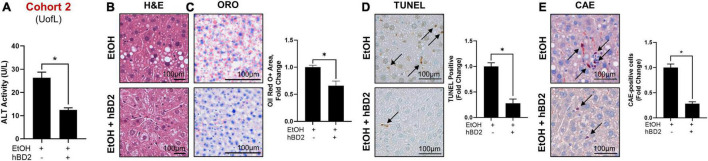
Human beta defensin 2 ameliorated liver injury in Cohort 2. **(A)** ALT activity levels. **(B–E)** Representative digital micrographs of H&E-stained liver sections, ORO-stained liver sections, TUNEL-stained liver sections, and CAE-stained liver sections with quantitation of ORO+ area, TUNEL+ cells, and CAE+ neutrophils shown to the right of each set of images, respectively. H&E images were captured at 100 × magnification; all other images were captured at 200 × magnification. Scale bar represents 100 μm in each panel. Data are reported as mean ± standard error of the mean. *p < 0.05.

Human beta defensin 2 administration significantly attenuated EtOH-induced liver injury in Cohort 1 as shown by reduced ALT levels in EtOH + hBD-2-treated mice compared to EtOH-fed mice (Figure 2A) with a slight decrease in liver steatosis as reflected in H&E staining and as assessed by ORO staining (Figures 2B,C). hBD-2 also reduced EtOH-induced hepatocyte cell death (Figure 2D) and modestly decreased hepatic neutrophil infiltration (Figure 2E). As in Cohort 1, ALT levels were significantly decreased in Cohort 2 EtOH + hBD-2 mice compared to EtOH-fed mice (Figure 3A). In contrast to Cohort 1, Cohort 2 mice had significant hBD-2-mediated attenuation of hepatic steatosis as shown by H&E staining and assessed by ORO staining (Figures 3B,C), as well as significantly decreased hepatocyte cell death (Figure 3D), and liver neutrophil infiltration (Figure 3E). Collectively, our data demonstrated that hBD-2 afforded a significant protection against experimental ALD in two independent cohorts of mice with distinct initial gut microbiota. When comparing the two Cohorts, hepatic steatosis and inflammation were attenuated to a greater extent in Cohort 2 mice.

### Intestinal Barrier Permeability Was Not Affected by Human Beta Defensin 2

Human beta defensin 2 had little impact on gut barrier permeability, as demonstrated by similar plasma LPS levels between groups in each cohort and between cohorts (Table 2). Similarly, mRNA expression of small intestine tight junction proteins *Zo1*, *Zo2*, and *Occludin* was unchanged between experimental groups in both cohorts (Table 3).

**TABLE 3:** Small intestine mRNA expression of tight junction proteins in PF or EtOH-fed control or hBD-2-treated mice.

	Cohort 1			Cohort 2	
	PF	EtOH	EtOH + hBD-2	EtOH	EtOH + hBD-2
	*n* = 6	*n* = 6	*n* = 6	*n* = 8	*n* = 10
*Zo-1*	1.040 ± 0.119	1.137 ± 0.090	1.367 ± 0.124	1.223 ± 0.279	0.840 ± 0.119
*Zo-2*	1.055 ± 0.136	1.162 ± 0.125	1.218 ± 0.159	1.509 ± 0.348	1.065 ± 0.180
*Occludin*	1.246 ± 0.342	1.295 ± 0.348	1.416 ± 0.372	1.553 ± 0.444	1.081 ± 0.225

*Values are expressed as mean Fold Change (vs. PF for Cohort 1 or EtOH for Cohort 2) ± SEM.*

### Human Beta Defensin 2 Led to Favorable Immunomodulatory Changes in a Cohort-Dependent Manner

**FIGURE 4:**
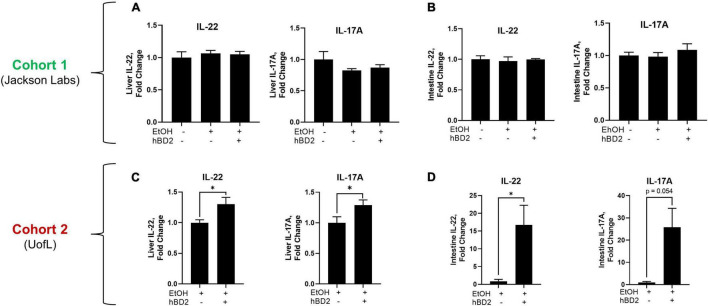
Human beta defensin 2 altered liver and small intestine immunomodulatory cytokines. Protein levels of IL-22 and IL-17A in the liver **(A,C)** and small intestine tissue **(B,D)** of Cohort 1 and Cohort 2 mice, respectively. Data are reported as mean ± standard error of the mean. *p < 0.05.

To determine the potential mechanisms underlying the beneficial effects of hBD-2 on EtOH-associated liver injury, we first analyzed the immunomodulatory effects of hBD-2, specifically focusing on well-known cytokines that play an important role in ALD pathogenesis and innate immunity. We measured the expression of liver and small intestine IL-22, a hepatoprotective cytokine which also exerts beneficial effects in the gut (Wang et al., 2014), and IL-17A, a cytokine with pleiotropic effects including gut microbial defense (Hong et al., 2017; Jensen et al., 2021). In Cohort 1, liver and intestinal IL-22 and IL-17A protein levels were unchanged following either EtOH feeding or hBD-2 administration (Figures 4A,B). However, Cohort 2 EtOH + hBD-2-treated mice, compared to EtOH alone, had a significant increase in both IL-22 and IL-17A levels in the liver and small intestine (Figures 4C,D). At the mRNA level, hBD-2 had limited effects on *Il22* and *Il17a* expression, as well as the expression of other pro-inflammatory cytokines, including *Cxcl1*, *Mcp1*, and *Mip2a* in both cohorts (Table 4).

**TABLE 4:** Liver and small intestine mRNA expression of immunomodulatory or pro-inflammatory cytokines in PF or EtOH-fed control or hBD-2-treated mice.

	Cohort 1			Cohort 2	
Gene	PF	EtOH	EtOH + hBD-2	EtOH	EtOH + hBD-2
	*n* = 6	*n* = 6	*n* = 6	*n* = 8	*n* = 10
**Immunomodulatory cytokines**					
Liver *Il-17a*	1.095 ± 0.176	0.890 ± 0.150	0.813 ± 0.107	1.242 ± 0.241	1.860 ± 0.731
Liver *Il-22*	1.854 ± 0.786	1.672 ± 0.549	1.454 ± 0.781	1.491 ± 0.925	1.370 ± 0.528
Small intestine *Il-17a*	1.064 ± 0.085	0.999 ± 0.225	1.417 ± 0.141	1.102 ± 0.181	1.245 ± 0.209
Small intestine *Il-22*	1.118 ± 0.217	1.244 ± 0.421	0.559 ± 0.128	2.728 ± 1.369	13.440 ± 4.409
**Pro-inflammatory cytokines**					
Liver *Cxcl1*	1.185 ± 0.255	4.393 ± 1.173	3.504 ± 0.481	0.792 ± 0.105	0.740 ± 0.163
Liver *Mcp1*	1.638 ± 0.398	2.307 ± 0.357	2.068 ± 0.391	0.959 ± 0.211	0.719 ± 0.079
Liver *Mip2α*	1.273 ± 0.340	0.652 ± 0.151	0.422 ± 0.065	1.121 ± 0.224	0.690 ± 0.063

*Values are expressed as mean Fold Change (vs. PF for Cohort 1 or EtOH for Cohort 2) ± SEM.*

Taken together, the differences in IL-22 and IL-17A expression between cohorts (each were elevated in Cohort 2 and unchanged in Cohort 1) support cohort-specific mechanisms contributing to hBD-2-mediated attenuation of EtOH-associated liver injury.

### Human Beta Defensin 2 Increased Intestine and Liver T Regulatory Cell Populations

**FIGURE 5:**
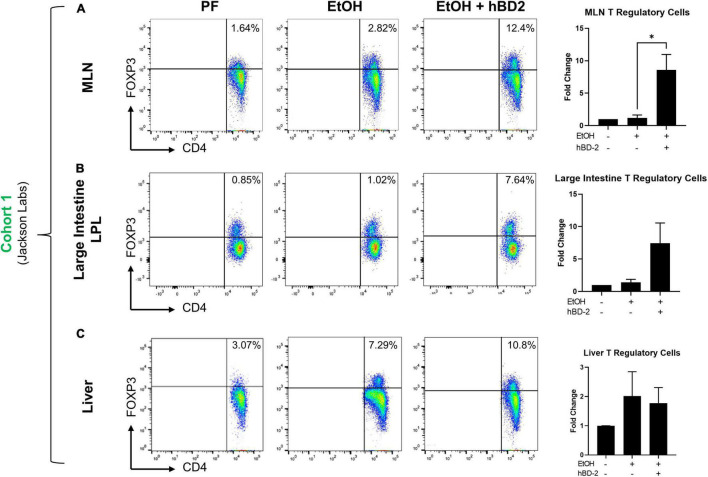
Gut immunomodulatory effects of hBD-2 on Treg populations. Relative abundance of Tregs in EtOH-fed mice relative to pair-fed mice was determined in the mesenteric lymph nodes **(A)**, large intestine lamina propria **(B)**, and liver **(C)** in Cohort 1 animals. For each tissue, representative dot plots for each group are shown on the left (as determined by distance from mean), with percentages of FOXP3-positive cells displayed in the upper-right-hand quartile, and overall quantitation for the whole group shown on the right. Data are reported as mean ± standard error of the mean. *p < 0.05.

Considering the immune regulatory potential of hBD-2 by dendritic cell engagement (Koeninger et al., 2020), we next investigated whether immune cells which interact with dendritic cells were altered in hBD-2 treated mice, including CD4^+^ IFNγ^+^ Th1 and CD4^+^ IL17^+^ Th17 T helper cells, CD4^+^ FOXP3^+^ T regulatory cells (Tregs), and B220^+^ IgA^+^ and B220^+^ IgM^+^ B cells. We analyzed these cell populations in the mesenteric lymph nodes (MLN), lamina propria, intraepithelial large and small intestine compartments, and liver of Cohort 1 mice (workflow and gating strategy is described in Supplementary Figure 1). We found that in the MLNs, hBD-2 caused a significant, ∼8.5-fold increase in Treg abundance relative to EtOH-fed mice (Figure 5A), indicating a distinct shift toward an immune-resolving profile. In the large intestine lamina propria, hBD-2 also caused an increase in the Treg population (Figure 5B), although this trend did not reach statistical significance. In the liver, hBD-2 did not increase Treg abundance (Figure 5C), indicating a gut-specific effect. In addition, there were no significant effects of hBD-2 on the abundance of Th1/Th17 cells in the MLN, large intestine lamina propria, or liver, and IgA^+^ and IgM^+^ B cells in the MLN and small intestine lamina propria, despite some nominal trends (Supplementary Figures 2A,B, respectively).

### Human Beta Defensin 2 Shifted the Composition of the Gut Microbiota Toward a Pair Fed-Like Phenotype

**FIGURE 6:**
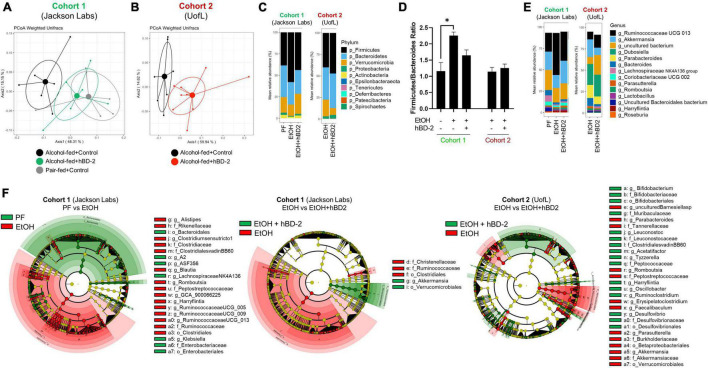
Human beta defensin 2 alters the composition of the gut microbiota. **(A,B)** Principal coordinate analysis using weighted UniFrac distance at the conclusion of the study for Cohort 1 and 2, respectively. Individual data points are shown as points with indicated group mean and 50% confidence interval. PERMANOVA between EtOH and EtOH + hBD-2 *p* = 0.037 and *p* = 0.002 in panels **(A,B)**, respectively. **(C)** Phylum-level summary of mean relative bacterial abundance. **(D)** *Firmicutes*/*Bacteroides* ratio. Data are reported as mean ± standard error of the mean. *p < 0.05. **(E)** Genus-level summary of mean relative bacterial abundance. **(F)** LEfSE cladograms showing taxonomical groups of bacteria associated PF or EtOH conditions in Cohort 1 (green and red, respectively; left), EtOH or EtOH + hBD-2 conditions in Cohort 1 (green and red, respectively; middle), and EtOH or EtOH + hBD-2 conditions in Cohort 2 (green and red, respectively; right).

Since microbial imprinting is a major mechanism of AMPs, we next investigated whether hBD-2 administration altered the luminal gut microbiota in our two cohorts. Chronic EtOH feeding alone led to significant changes in gut microbiota, as apparent when comparing pair-fed (PF) and EtOH-fed mice in Cohort 1, which separated into distinct clusters by PCoA (Figure 6A). hBD-2, however, shifted the microbial composition in EtOH-fed mice toward a PF-like phenotype resulting in a clear separation of EtOH + hBD-2-treated mice from EtOH alone-treated mice in Cohort 1 (Figure 6A), as well as in Cohort 2 (Figure 6B), indicating a strong effect of hBD-2 on the EtOH-induced perturbation of the gut microbiota in both cohorts. Analysis of major phyla and genera revealed that in both study cohorts, Firmicutes, Bacteroides, and Verrucomicrobia constituted the most abundant phyla regardless of EtOH or hBD-2 treatment (Figure 6C). The ratio between Firmicutes and Bacteroides, an indicator of pathological changes in the gut microbiota (Stojanov et al., 2020), was increased by EtOH in Cohort 1 (Figure 6D), primarily due to an increase in Firmicutes. This increase was attenuated by hBD-2. Interestingly, in Cohort 2, the Firmicutes/Bacteroides ratio was relatively unchanged by hBD-2. At the genus level, in both cohorts, *Ruminococcaceae*, *Akkermansia*, and *Bacteroides* constituted the most abundant genera, whereas *Dubosiella* and *Parabacteroides* were prominent genera in Cohort 2 only (Figure 6E).

Next, we sought out specific taxonomical groups that were enriched by EtOH or hBD-2 administration (cladograms, Figure 6F). Taxonomical groups that were enriched by EtOH feeding included orders Betaproteobacteriales, Verrucomicrobiales, and Clostridiales, as well as several families including *Tannerellaceae*, *Burkholderiaceae*, *Akkermansiaceae*, *Rikenellaceae*, *Clostridiaceae*, *Ruminococcaceae*, and *Christensenellaceae*. In Cohort 1, relative to EtOH-fed mice, PF mice were enriched for orders Bacteroidales and Enterobacteriales, as well as family *Enterobacteriaceae*. In this cohort, hBD-2 enriched order Verrucomicrobiales and genus *Akkermansia*. hBD-2 also enriched several taxonomical groups in Cohort 2 including orders Desulfovibrionales and Bifidobacteriales as well as families *Peptococcaceae*, *Leuconostocaceae*, *Muribaculaceae*, and *Clostridialesvadin BB60*. When adjusting for differences in microbiota between the two cohorts *via* linear mixed-effects modeling, we further identified that numerous amplicon sequence variants (ASVs) within the family *Ruminococcaceae* were altered by hBD-2, indicating strong effects within this group (Table 5). Despite these varied EtOH and hBD-2 effects, neither condition changed overall alpha diversity by multiple indices (Supplementary Figures 3A–C).

**TABLE 5:** Linear mixed-effects modeling identified bacterial genera which were altered by hBD-2 in both cohorts adjusted for cohort-specific differences.

Genus	*P* value
g__*Bacteroides*	0.0443
g__*Desulfovibrio*	0.0250
g__*Dubosiella*	0.0245
g__*Dubosiella*	0.0400
g__*Dubosiella*	0.0425
g__*Faecalibaculum*	0.0497
g__*Parabacteroides*	0.0099
g__*Parabacteroides*	0.0299
g__*Rikenellaceae RC9 gut group*	0.0325
g__*Ruminiclostridium 9*	0.0148
g__*Ruminococcaceae UCG-009*	0.0453
g__*Ruminococcaceae UCG-013*	0.0201
g__*Ruminococcaceae UCG-013*	0.0257
g__*Ruminococcaceae UCG-013*	0.0312
g__*Ruminococcaceae UCG –013*	0.0320
g__*Ruminococcaceae UCG-013*	0.0394
g__*Ruminococcaceae UCG-013*	0.0403
g__*Ruminococcaceae UCG-013*	0.0417
g__*Ruminococcaceae UCG-013*	0.0433
g__*Ruminococcaceae UCG-013*	0.0446
g__*Ruminococcaceae UCG-013*	0.0474
g__*Ruminococcaceae UCG-013*	0.0489

**FIGURE 7:**
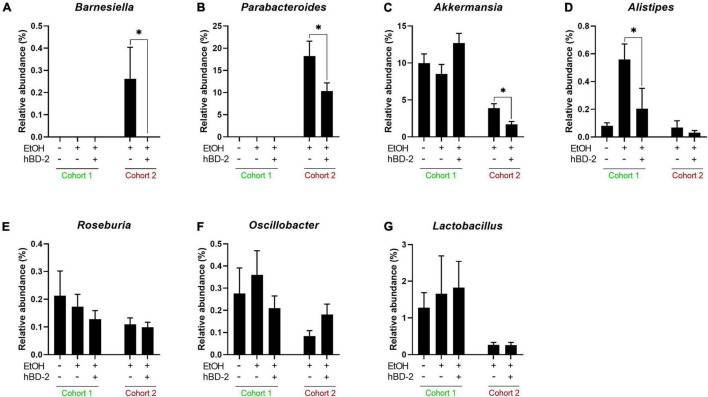
Genus-level effects of hBD-2. Relative abundance (%) is shown for seven genera of interest in EtOH-associated liver disease, specifically, *Barnesiella*, *Parabacteroides*, *Akkermansia*, *Alistipes*, *Roseburia*, *Oscillobacter*, and *Lactobacillus* (**A–G**, respectively). Data are reported as mean ± standard error of the mean. *p < 0.05.

Lastly, we analyzed changes in abundance of several major genera with well characterized roles in ALD (Figure 7). hBD-2 led to a significant reduction in abundance of *Barnesiella*, *Parabacteroides*, and *Akkermansia* in our Cohort 2 mice (Figures 7A–C, respectively). Similarly, genus *Alistipes* was significantly decreased by hBD-2 in our Cohort 1 mice (Figure 7D). Another genus, *Roseburia*, was unaffected by EtOH and hBD-2 in both cohorts (Figure 7E). Additionally, these data revealed several differences between cohorts, specifically, an absence of *Barnesiella* and *Parabacteroides* in Cohort 1 (Figures 7A,B). The abundance of *Oscillobacter* and *Lactobacillus* was not affected in any group in both cohorts (Figures 7F,G).

## Discussion

**FIGURE 8:**
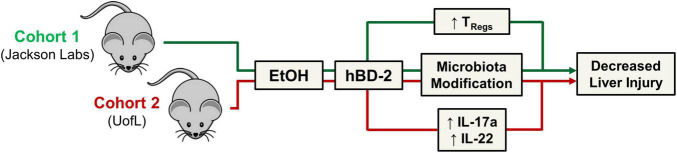
Summary figure. Schematic representation of study findings. hBD-2 ameliorated experimental alcohol-associated liver disease in two independent cohorts of mice with distinct gut microbiota.

In the current study, we demonstrated the beneficial effects of hBD-2 in an experimental model of chronic ALD (as summarized in Figure 8). hBD-2, administered orally on each of the last 7 days of the chronic EtOH feeding, attenuated liver injury as evidenced by a significant reduction in ALT levels. Our observation that hBD-2 is protective in ALD is in line with previous studies demonstrating that other AMPs such as CRAMP and alpha defensins ameliorate alcohol-associated liver injury, steatosis, and inflammation (Li et al., 2020; Zhong et al., 2020). Importantly, our results showed that the beneficial effects of hBD-2 on EtOH-induced liver injury were independent of baseline host gut microbiota. We observed that hBD-2 had a unified protection against liver injury in two independent cohorts of mice (Cohort 1, vendor purchased, and Cohort 2, bred in-house) which had significant differences in baseline gut microbiota. These differences did, however, lead to distinct mechanisms governing the protective effects of hBD-2 against liver injury.

Indeed, while both cohorts were protected from liver injury, mechanistically, Cohort 2 mice had more profound changes in immunomodulatory cytokine levels. Specifically, hBD-2 administration in these mice led to significantly elevated levels of liver and intestinal IL-17A and IL-22. Interestingly, whereas IL-17A is typically considered a pro-inflammatory cytokine within the context of ALD (Lemmers et al., 2009), its expression is linked to increased anti-microbial peptide production, including defensins (Iwakura et al., 2008). IL-17 upregulates hBD-2 in the airway epithelium in a JAK/NFκB-mediated mechanism (Kao et al., 2004), suggesting a potential positive-feedback loop. hBD-2 also increased small intestine and liver IL-22 levels in our Cohort 2 mice. IL-22 is recognized as a beneficial cytokine in ALD (Ki et al., 2010; Sabat et al., 2014; Wang et al., 2014; Gulhane et al., 2016; Arab et al., 2020; Hwang et al., 2020; Xiang et al., 2020). Numerous reports demonstrate IL-22-mediated protection against alcohol-associated liver injury *via* upregulation of antioxidant and anti-apoptotic genes, downregulated lipogenesis, and increased liver regeneration and hepatocyte proliferation following injury (Ki et al., 2010; Hendrikx et al., 2019; Liu et al., 2020; Xiang et al., 2020). IL-22 also plays a defensive role in the gut mucosa against gram-negative microbes (Aujla and Kolls, 2009). hBD-2 may induce the expression of IL-17A and IL-22 through ROR-γt, a transcription factor which classically promotes Th17 differentiation (Ivanov et al., 2006) but has also been recently linked to Th22 differentiation (Plank et al., 2017; Sekimata et al., 2019), leading to downstream production of IL-17A and IL-22 by these T helper cell subtypes, respectively.

Whereas gut and liver IL-17A and IL-22 were unaffected by hBD-2 in Cohort 1, we did observe increased Treg abundance in these mice. Tregs are an immune-resolving T cell subset that plays a key role in a number of liver diseases (Van Herck et al., 2019), and is reduced in the liver in non-alcoholic fatty liver disease (Ma et al., 2007) and alcohol-associated hepatitis (Almeida et al., 2013). While we observed no difference in liver Tregs, we did notice a significant increase in mesenteric lymph node Tregs and a non-significant increase in large intestine Tregs. Gut Tregs may indirectly contribute to the attenuation of liver injury by maintaining gut homeostasis, preventing excess immune cell activation, and importantly, promoting gut barrier integrity *via* production of IL-10 (Corthay, 2009), although this mechanism needs to be further investigated. Further, while the exact mechanism by which hBD-2 increased gut Treg abundance is unclear, gut microbiota may have contributed to this increase, as some bacteria such as *Bacteroides fragilis* and *Clostridium rhamnosus* can induce the differentiation of some Treg phenotypes (Pandiyan et al., 2019). Apart from gut microbiota imprinting, a recent report elegantly demonstrated how CCR2 agonism enhances CD25 expression by FoxP3^+^ Tregs, thereby boosting their abundance (Zhan et al., 2020). Because hBD-2 curbs LPS-induced inflammation by CCR2 engagement (Koeninger et al., 2020), it seems possible that hBD-2 may potentially interact directly with gut Tregs *via* Treg-expressed-CCR2 to boost their abundance in the draining mesenteric lymph nodes. These results collectively suggest hBD-2 may function to modulate host immunity by upregulating beneficial cytokines and modulating Treg abundance to shift the liver and gut toward a less pro-inflammatory environment. This is in line with the growing concept that AMPs can act as signaling molecules with beneficial immunomodulatory functions, a role which beta defensins are thought to have acquired over evolutionary time (Meade and O’Farrelly, 2018). It is also possible that hBD-2 may function through other beneficial immunomodulatory effects. For example, hBD-2 was shown to enhance recruitment of circulating immature dendritic cells and memory T cells to sites of inflammation, improve antigen presentation *via* formation of defensin-antigen complexes to deliver antigens to immune cells (Yang et al., 2002; Auvynet and Rosenstein, 2009), and prevent toll like receptor-dependent pro-inflammatory pathway activation in dendritic cells (Koeninger et al., 2020).

While immunomodulation is increasingly recognized as a mechanism of action for AMPs, anti-microbial activity and gut microbiome modification is widely accepted as the primary mechanism for these molecules (Mahlapuu et al., 2016), as supported by several previous studies showing altered gut microbiota following AMP treatment (Li et al., 2020; Zhong et al., 2020). Similarly, in each of our cohorts, hBD-2 changed the composition of the luminal gut microbiota on multiple taxonomical levels, including the phylum, genus, and species levels. In Cohort 2, hBD-2 caused a decrease in abundance of bacteria within the genera *Parabacteroides* and *Barnesiella*, two gram-negative taxonomical groups within the phylum Bacteroides. While this phylum is generally considered beneficial in ALD (Ferrere et al., 2017), some evidence points to Bacteroides as a contributor to colonic inflammation in ulcerative colitis (Shen et al., 2018). We also noted that hBD-2 decreased the EtOH-induced rise in the Firmicutes/Bacteroides ratio, an indicator of gut microbial dysbiosis. Within phylum Firmicutes, hBD-2 had a distinct modulating effect on numerous ASVs within the *Ruminococcaceae* family. Bacteria within this genus are generally thought to be beneficial in ALD, as both mice fed alcohol and patients with alcohol-associated cirrhosis have decreased *Ruminococcaceae* (Hartmann et al., 2015; Bajaj, 2019). Transplant of fecal material rich in *Ruminococcaceae* and *Lachnospiraceae* into patients with alcohol use disorder has been shown to decrease alcohol craving in a phase I clinical trial (Bajaj et al., 2020). *Ruminococcaceae* is also a major producer of the short-chain fatty acid butyrate (Vital et al., 2017), a beneficial molecule which attenuates liver injury and improves barrier function in experimental ALD (Cresci et al., 2017) and may potentially contribute to the beneficial effects of hBD-2 in our model.

Apart from these mechanistic effects of hBD-2, the question remains whether the beneficial effects of this peptide in the liver were mediated directly (i.e., host cell receptor-mediated *via* CCR2 or others) or indirectly (i.e., *via* the gut microbiota). Whether hBD-2 can cross the intestinal barrier to exert direct effects on the liver currently remains largely unknown. Potentially, in disease states with increased intestinal permeability, including ALD (Szabo, 2015), hBD-2 may be able to translocate to the systemic circulation. However, in general, peptides are considered to have low bioavailability after intestinal absorption due to rapid proteolytic cleavage (Diao and Meibohm, 2013). Despite this general assumption of diminished pharmacokinetics, it is worth noting that both intranasal and oral administration of hBD-2 alleviated experimental asthma (Borchers et al., 2021; Pinkerton et al., 2021), just as systemic administration of this peptide mitigated gut inflammation in three different colitis models (Koeninger et al., 2020), corroborating the pronounced extraintestinal efficacy of hBD-2. Alternatively, assessing the efficacy of hBD-2 in gut microbiota-depleted mice may also help to address this question. However, microbiota-deficient mice, e.g., germ free mice, have altered immunity and ethanol metabolism which confound the protective effects of hBD-2 (Chen et al., 2015). A previous report regarding the use of another AMP, fungal lysozyme, showed that in an experimental colitis model the presence of gut microbiota was required for the therapeutic effects of the treatment (Larsen et al., 2021), suggesting that the effects of hBD-2 may similarly be microbiota-dependent.

Overall, our data showed the beneficial effects of hBD-2 administration in experimental ALD *via* mechanisms associated with gut and liver immunomodulation and gut microbiota modification. Our data benefit from the use of two independent cohorts of mice with distinct gut microbiota which both showed protection against chronic alcohol-induced liver injury, albeit by different mechanisms. Future studies should, however, address the efficacy of hBD-2 in other experimental models of ALD, given that the chronic EtOH feeding model used here recapitulates mostly early onset features of human ALD (Lamas-Paz et al., 2018). The ability of hBD-2 to ameliorate more severe liver injury, inflammation, and fibrosis remains undetermined. Future work should also further examine the role of specific bacterial taxa such as *Ruminococcaceae* in hBD-2-mediated benefits in ALD.

## Data Availability Statement

The datasets presented in this study can be found in online repositories. The names of the repository/repositories and accession number(s) can be found below: ENA, PRJEB49248.

## Ethics Statement

The animal study was reviewed and approved by University of Louisville Institutional Animal Care and Use Committee.

## Author Contributions

All authors have reviewed and approved the manuscript. IK and JW: conceptualization. DW, JW, YS, and IL: methodology. JW and IL: software, writing—original draft preparation, and visualization. JW, IL, BJ, IK, and DW: formal analysis. IK, JW, IL, BJ, DW, and JH: investigation. IK, CM, and BJ: resources and funding acquisition. IL: data curation. JW, JH, DW, IL, BJ, CM, and IK: writing—review and editing. IK and BJ: supervision. IK: project administration.

## Author Disclaimer

The content is solely the responsibility of the authors and does not necessarily represent the official views of the National Institutes of Health.

## Conflict of Interest

The authors declare that the research was conducted in the absence of any commercial or financial relationships that could be construed as a potential conflict of interest.

## Publisher’s Note

All claims expressed in this article are solely those of the authors and do not necessarily represent those of their affiliated organizations, or those of the publisher, the editors and the reviewers. Any product that may be evaluated in this article, or claim that may be made by its manufacturer, is not guaranteed or endorsed by the publisher.
