# Supplementary material for: Human Beta Defensin 2 Ameliorated Alcohol-Associated Liver Disease in Mice
Source: Front Physiol. 2022 Jan 27;12:812882. doi: 10.3389/fphys.2021.812882 (PMC8829467; doi:10.3389/fphys.2021.812882)
Supplement: Supplementary file 1 [file Presentation_1.PPTX]

## Slide 1
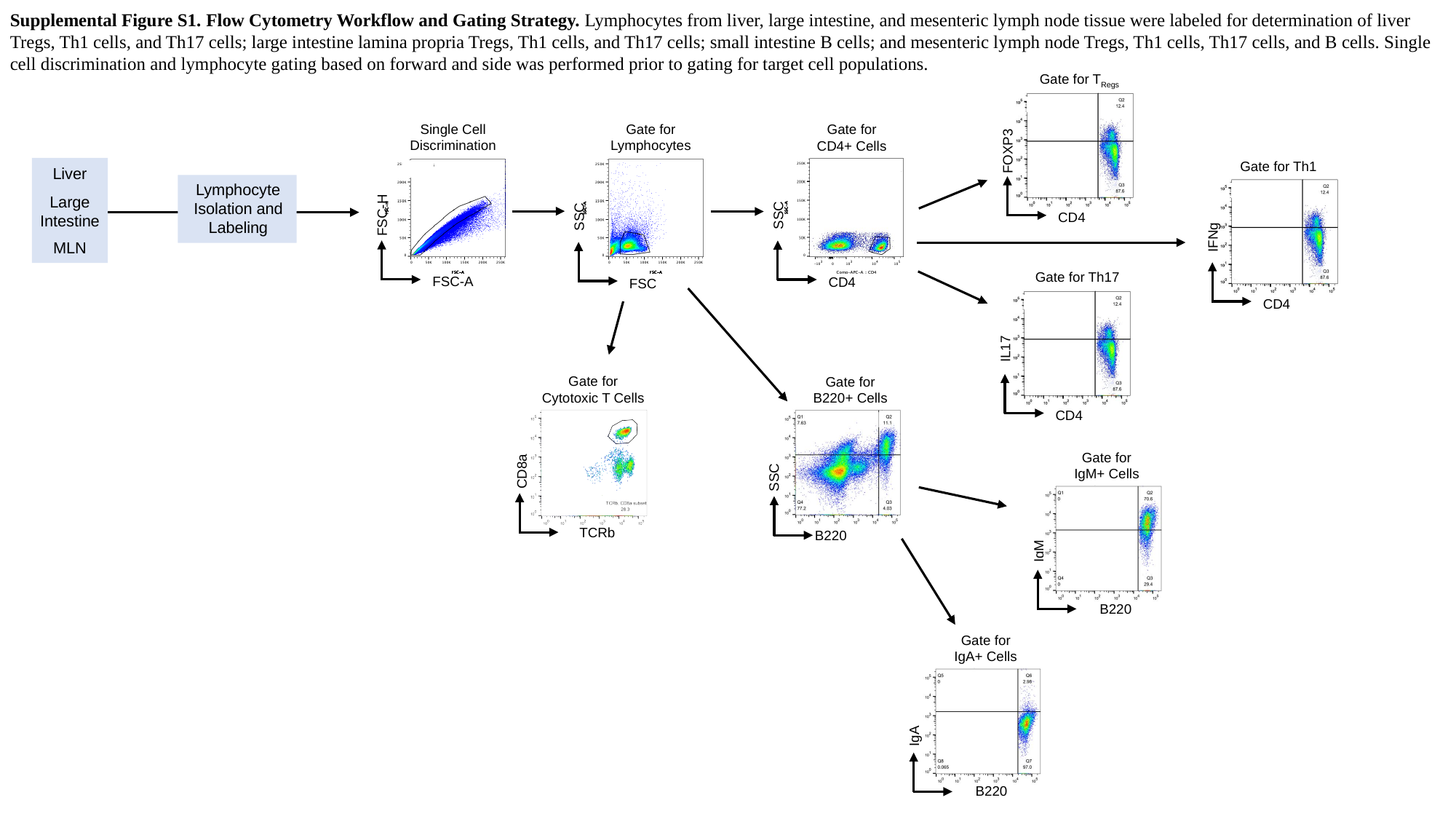

Supplemental Figure S1. Flow Cytometry Workflow and Gating Strategy. Lymphocytes from liver, large intestine, and mesenteric lymph node tissue were labeled for determination of liver Tregs, Th1 cells, and Th17 cells; large intestine lamina propria Tregs, Th1 cells, and Th17 cells; small intestine B cells; and mesenteric lymph node Tregs, Th1 cells, Th17 cells, and B cells. Single cell discrimination and lymphocyte gating based on forward and side was performed prior to gating for target cell populations.
Gate for TRegs
FOXP3
CD4
Single Cell Discrimination
Gate for Lymphocytes
Gate for CD4+ Cells
Gate for Th1
SSC
CD4
FSC-H
FSC-A
SSC
FSC
Liver
Large Intestine
MLN
Lymphocyte Isolation and Labeling
IFNg
CD4
Gate for Th17
IL17
CD4
Gate for Cytotoxic T Cells
Gate for B220+ Cells
SSC
B220
CD8a
TCRb
Gate for IgM+ Cells
IgM
B220
Gate for IgA+ Cells
IgA
B220

## Slide 2
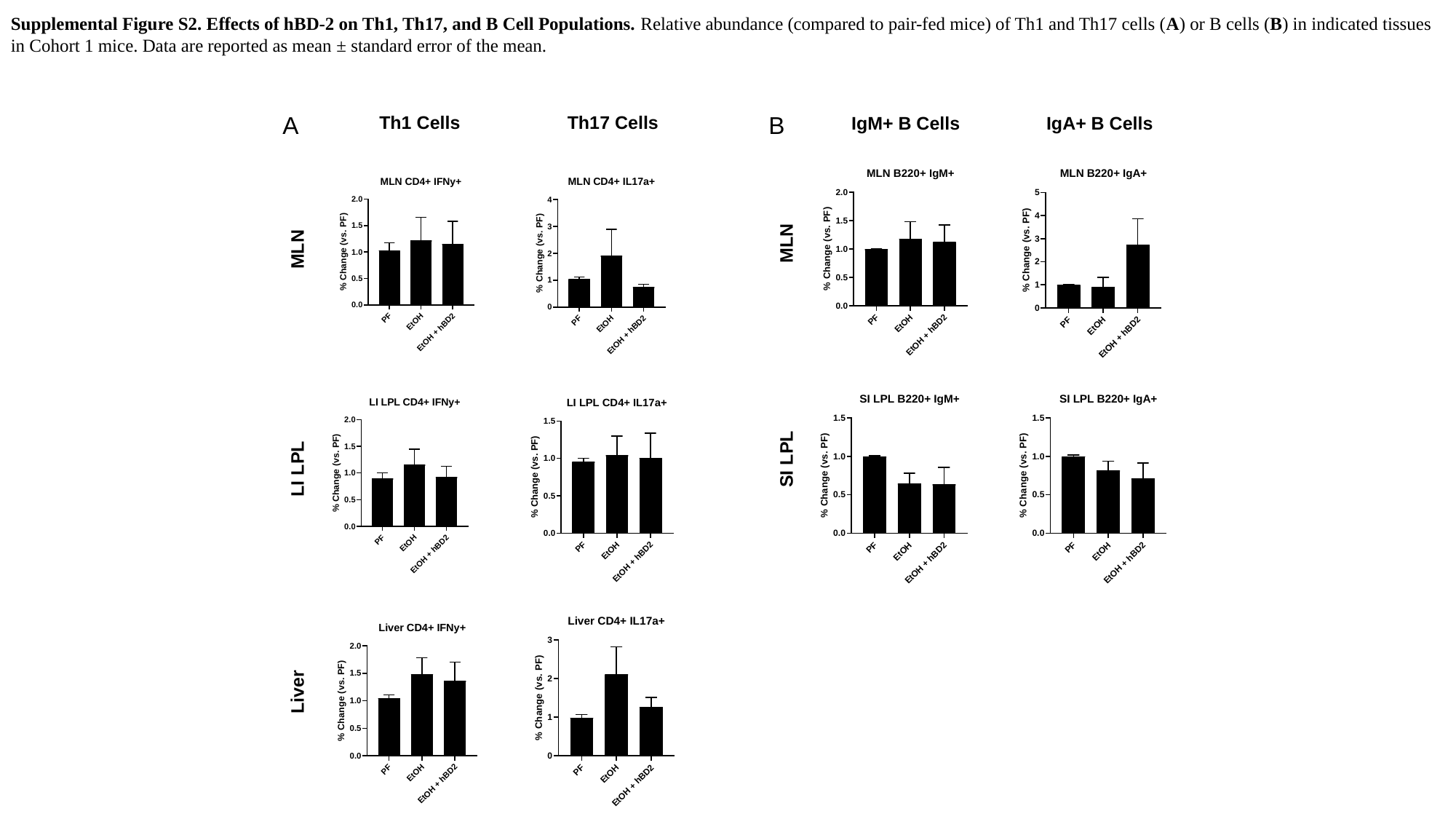

Supplemental Figure S2. Effects of hBD-2 on Th1, Th17, and B Cell Populations. Relative abundance (compared to pair-fed mice) of Th1 and Th17 cells (A) or B cells (B) in indicated tissues in Cohort 1 mice. Data are reported as mean ± standard error of the mean.
A
B
Th17 Cells
Th1 Cells
IgM+ B Cells
IgA+ B Cells
MLN
MLN
SI LPL
LI LPL
Liver

## Slide 3
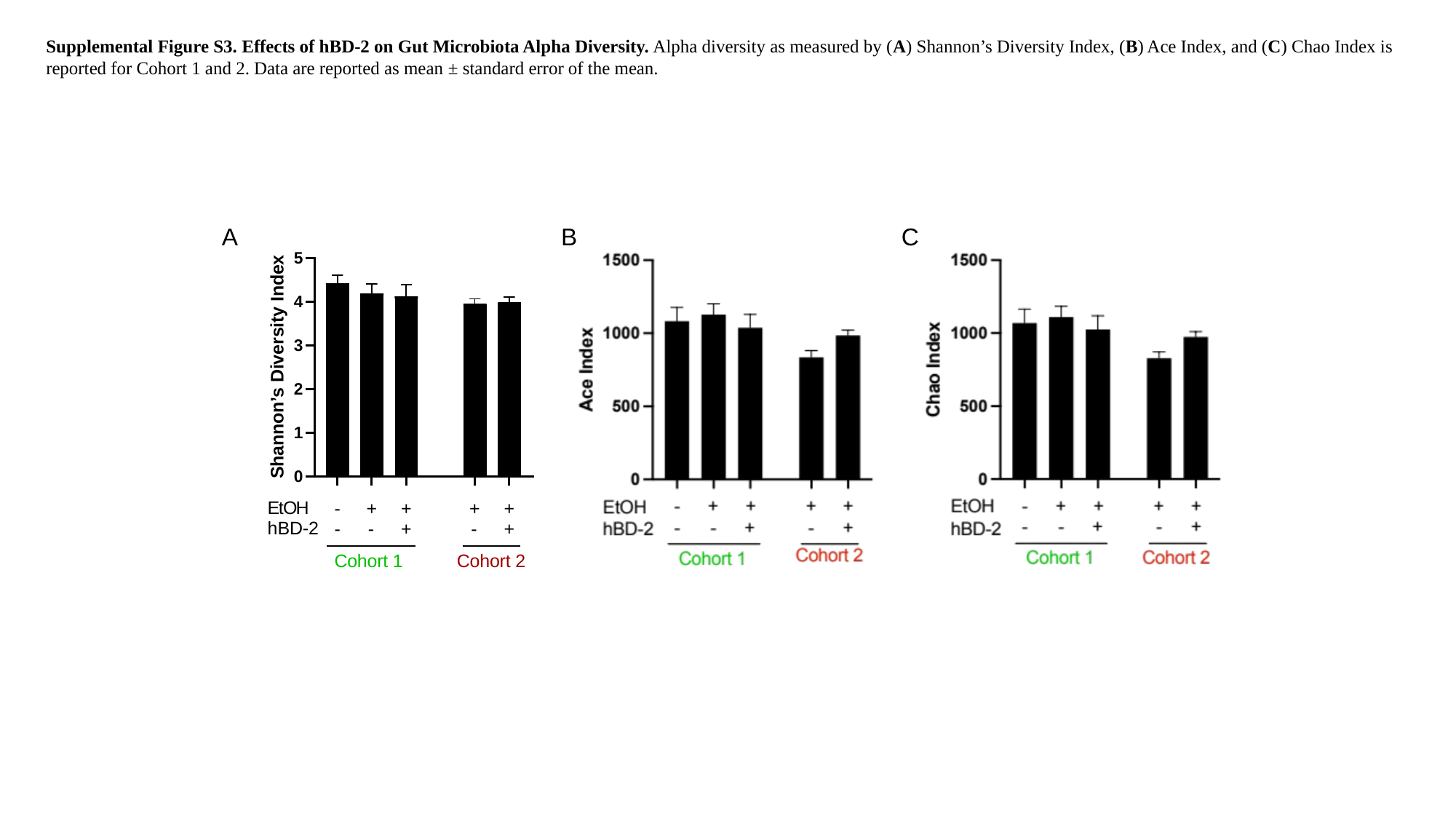

Supplemental Figure S3. Effects of hBD-2 on Gut Microbiota Alpha Diversity. Alpha diversity as measured by (A) Shannon’s Diversity Index, (B) Ace Index, and (C) Chao Index is reported for Cohort 1 and 2. Data are reported as mean ± standard error of the mean.
A
B
C
